# Translating economic evaluations into financing strategies for implementing evidence-based practices

**DOI:** 10.1186/s13012-021-01137-9

**Published:** 2021-06-29

**Authors:** Alex R. Dopp, Suzanne E. U. Kerns, Laura Panattoni, Jeanne S. Ringel, Daniel Eisenberg, Byron J. Powell, Roger Low, Ramesh Raghavan

**Affiliations:** 1 Department of Behavioral and Policy Sciences, RAND Corporation, 1776 Main Street, Santa Monica, CA 90401 USA; 2 Graduate School of Social Work, University of Denver, Craig Hall, 2148 South High St, Denver, 80208 CO USA; 3 The Kempe Center for the Prevention and Treatment of Child Abuse and Neglect, University of Colorado, 13123 E 16th Ave, Aurora, CO 80045 USA; 4 Hutchinson Institute for Cancer Outcomes Research, Fred Hutchinson Cancer Research Center, 1100 Fairview Ave N, Seattle, WA 98109 USA; 5 Department of Economics, Sociology, and Statistics, RAND Corporation, 1776 Main Street, Santa Monica, CA 90401 USA; 6 Fielding School of Public Health, University of California Los Angeles, 650 Charles E Young Dr S, Los Angeles, CA 90095 USA; 7 Department of Psychiatry and Behavioral Sciences, University of California Los Angeles, 757 Westwood Plaza #4, Los Angeles, CA 90095 USA; 8 Brown School and School of Medicine, Washington University in St. Louis, Campus Box 1196, One Brookings Drive, St. Louis, MO 63130 USA; 9 America Forward, 1400 Eye St. NW, Suite 900, Washington, DC 20005 USA; 10 Silver School of Social Work, New York University, 1 Washington Square North, Room 301, New York, NY 10003 USA

**Keywords:** Economic evaluation, Cost-effectiveness, Cost-benefit, Healthcare financing, Implementation strategies

## Abstract

**Background:**

Implementation researchers are increasingly using economic evaluation to explore the benefits produced by implementing evidence-based practices (EBPs) in healthcare settings. However, the findings of typical economic evaluations (e.g., based on clinical trials) are rarely sufficient to inform decisions about how health service organizations and policymakers should finance investments in EBPs. This paper describes how economic evaluations can be translated into policy and practice through complementary research on financing strategies that support EBP implementation and sustainment.

**Main body:**

We provide an overview of EBP implementation financing, which outlines key financing and health service delivery system stakeholders and their points of decision-making. We then illustrate how economic evaluations have informed decisions about EBP implementation and sustainment with three case examples: (1) use of Pay-for-Success financing to implement multisystemic therapy in underserved areas of Colorado, USA, based in part on the strength of evidence from economic evaluations; (2) an alternative payment model to sustain evidence-based oncology care, developed by the US Centers for Medicare and Medicaid Services through simulations of economic impact; and (3) use of a recently developed fiscal mapping process to collaboratively match financing strategies and needs during a pragmatic clinical trial for a newly adapted family support intervention for opioid use disorder.

**Conclusions:**

EBP financing strategies can help overcome cost-related barriers to implementing and sustaining EBPs by translating economic evaluation results into policy and practice. We present a research agenda to advance understanding of financing strategies in five key areas raised by our case examples: (1) maximize the relevance of economic evaluations for real-world EBP implementation; (2) study ongoing changes in financing systems as part of economic evaluations; (3) identify the conditions under which a given financing strategy is most beneficial; (4) explore the use and impacts of financing strategies across pre-implementation, active implementation, and sustainment phases; and (5) advance research efforts through strong partnerships with stakeholder groups while attending to issues of power imbalance and transparency. Attention to these research areas will develop a robust body of scholarship around EBP financing strategies and, ultimately, enable greater public health impacts of EBPs.

Contributions to the literature

- We describe the need for implementation research to inform financing strategies, which secure and direct funds to support EBP adoption and continued use.
- We articulate an important gap between economic evaluation findings from research and the development of financing strategies in practice and policy.
- Our overview of EBP financing identifies the roles and relevant decisions for key health service system stakeholders.
- Our case examples highlight how different EBP financing strategies have been used to translate data on economic impact into practice and policy.
- We propose a research agenda for advancing the complementary impact of economic evaluations and financing strategies.

## Background

Implementation research often presupposes that increased use of evidence-based practices (EBPs) will produce improved health and associated benefits for patients, health systems, and society. Researchers are increasingly using economic evaluations to empirically compare costs and outcomes between discrete clinical scenarios—including use of EBPs and associated implementation strategies [1–3]. Yet there is little guidance for policymakers and leaders of health service organizations regarding how to use findings from economic evaluations to optimize EBP implementation and sustainment outcomes at a reasonable cost. This paper provides an orientation to EBP financing and presents three case examples that demonstrate how policymakers and health service organizations can apply research findings about the economics of EBPs and implementation strategies in policy and practice.

Such translation is important, given that the added costs of implementing and sustaining EBPs are a barrier to their use in healthcare settings [4–6]. Balancing costs and benefits of various healthcare practices is fundamental to achieving the greatest benefits to the largest number of patients at the lowest per-patient cost [7]. Therefore, many scholars have called for more frequent and higher quality use of economic evaluation in implementation research [8–10], and articles in this special collection describe a path forward. Today, economic evaluations use a variety of analyses (e.g., cost-benefit, cost-effectiveness, budget impact) but are all meant to provide structured support to decision-makers regarding the relative costs and benefits of multiple, distinct courses of action [11, 12] (e.g., “Which clinical practice will maximize patient health benefits for the money spent?”, “Which strategy will optimize implementation of this EBP within current budget constraints?”). Often, these courses of action have been defined and compared within a clinical trial to determine which produces the greatest benefits for the money spent from a given perspective (e.g., societal, health system). The challenges of generalizing from clinical trials to practice and policy are well-documented and include (but are not limited to) non-representative samples and settings, extensive resources for measuring implementation and outcomes, and limited ability to evaluate rare or long-term events [13–15].

Much as efficacy findings about EBPs rarely change clinical practice without concerted implementation efforts, economic evaluation findings alone are unlikely to change large-scale patterns of investment in EBPs. A major remaining challenge—and the focus of this paper—is determining *how* to finance EBPs and implementation strategies. Research from varied global contexts consistently shows that economic evaluations are one of many important factors in decision-making about EBP use [16–18]. Financing the implementation and sustainment of EBPs might maximize return on investment, but implementation researchers need to start considering who should pay for the services and by what means they should provide the money. In economic evaluations, societal costs and benefits are often aggregated across various actors (e.g., patients, health service organizations, government agencies, taxpayers) and across fixed and variable costs [19]. In what has been termed the “wrong pockets” problem [20], the social and economic benefits of the EBP accumulate over long periods of time and appear in other sectors, raising questions about which sectors should fund implementation. Coupled with the fact that variable implementation costs (e.g., training and consultation, measurement-based care) are often not covered by traditional service-focused financing options like health insurance [21, 22], the pathway to financing an EBP or implementation strategy becomes even less straightforward. Ongoing work is making economic evaluations more relevant to implementation (see [9])—e.g., through increased use of budget impact analyses to characterize budget gaps—but additional information is needed by key healthcare delivery and financing decision-makers.

Maximizing the public health impact of EBPs requires strategies that align service delivery and financing to effectively support and sustain implementation [23]. Such strategies remain under-researched, but a recent scoping review compiled the first dedicated list of “financing strategies” that support EBP implementation and sustainment [24]. The authors defined 23 potential financing strategies and characterized their frequency of use in health services research. Examples of currently used financing strategies included enhanced fee-for-service reimbursement rates (with the additional reimbursement covering implementation costs) and the use of grants, contracts, and budget line-items to fund EBP adoption. The review focused on behavioral health services, but also documented usage in health and social services more broadly [24], and its findings and recommendations were consistent with those from a systematic review of alternative payment models for cancer care [25]. The authors noted a lack of rigorous research on the role of financing strategies within EBP implementation and recommended that implementation scientists begin investigating these strategies.

The present paper describes how economic evaluations and financing strategies can together inform EBP implementation within healthcare policy and practice. We begin with an overview of financing for EBP implementation, highlighting key decisions involved for stakeholders. We then present three case examples depicting how the results of economic evaluation can inform those decisions—and the limitations encountered. We conclude by articulating a research agenda in which investigations of economic impact and financing strategies can mutually advance large-scale investment in EBPs.

## Overview of EBP implementation financing

**Fig. 1:**
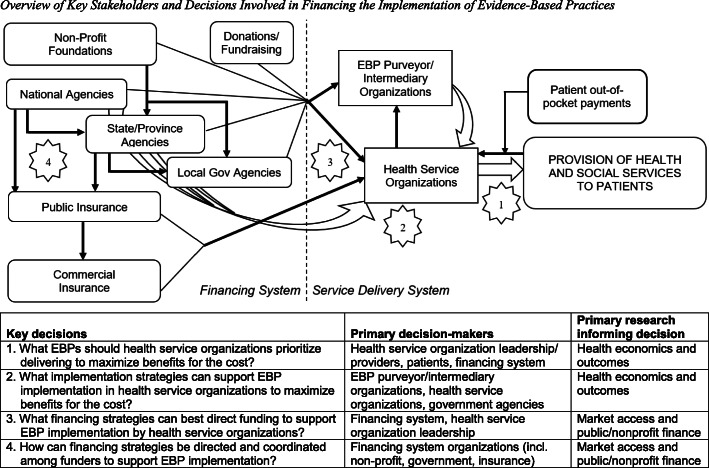
Overview of key stakeholders and decisions involved in financing the implementation of evidence-based practices. *Note.* EBP, evidence-based practice. Black arrows represent cash flows; straight white arrows represent delivery of health and social services (including EBPs); curved white arrows represent delivery of non-financial implementation strategies. For simplicity, adjacent arrows of the same type are sometimes merged in the figure, but this does not imply the activities of those organizations are coordinated. The numbers represent four key decisions made when financing the implementation of EBPs, which are detailed below the figure

Adoption and continued use of EBPs is challenging due to the complex and fragmented methods of financing health services [21–23]. Figure 1 summarizes the key stakeholders and decisions involved in financing EBP implementation. The figure depicts two interlinked systems: (1) The *service delivery* system involves direct provision of health services (including EBPs) and includes health service provider organizations (e.g., clinics, hospitals, service centers) and the patients they serve. Also within this system, health service organizations may receive non-monetary support and guidance in using EBPs from purveyor/intermediary organizations (some general and some EBP-specific) [26]. (2) The *financing* system is made up of various agencies that provide funding to health service organizations in exchange for delivering care. Examples of funders include government agencies (national, state/province, local) that support services directly or through related research, non-profit foundations, donors, and insurance plans (public or commercial). Each funder may provide financing to health service organizations and/or engage in complex interactions with other funders (e.g., pass-through funds from national to state/local governments, managed care organizations that administer public insurance plans). Altogether, payments to providers and provider organizations from funding agencies account for most healthcare expenditures (e.g., 87% in the USA [21, 22]); thus, funders can influence service delivery system activities through the incentives embedded in their payment models. Moreover, many government agencies (e.g., health services, behavioral health, social services) also provide non-financial implementation support, such as training or technical assistance, to health service organizations. Note that we used generic terminology so Fig. 1 could apply to financing systems in various countries, although the relevance of each component will vary by country.

Figure 1 details four decision points involved in financing EBPs in healthcare services. These decisions cross levels of administration and policy, making them challenging for stakeholders to coordinate. They also differ in how directly they are informed by research on (a) health economics and outcomes, principally economic evaluations, versus (b) market access, which considers issues of efficiency in service delivery—including how best to pay for an innovation [27]. Decisions 1 and 2 relate to the selection of EBPs and implementation strategies that will maximize patients’ service outcomes. Economic evaluations of implementation create a strong foundation for such decisions. Market access becomes central when financing decisions are required for new activities—i.e., how funding agencies should finance the EBPs and implementation strategies (decision 3) and coordinate with each other (decision 4). Research on financing public and non-profit private organizations more broadly is also helpful to consider for these decisions; for example, studies demonstrate the benefits of having diverse revenue streams [28]. If the funding system cannot offer adequate financing strategies, then the service delivery system may never successfully execute its EBP implementation decisions [24], even when economic evaluations suggest considerable benefits would result.

## Case examples

The financing of EBP implementation—and the role of economic evaluation therein—is highly complex, as shown in Fig. 1 and the discussion thus far. Next, we present three case examples of EBP financing to illustrate the topic in a more concrete manner; narratives demonstrating how complex EBP financing decisions have drawn on economic evaluation results can be powerful for decision-makers. Table 1 summarizes these examples, which represent a diverse range of service sectors, clinical problems, EBPs, and financing strategies. The table notes the financing stakeholders and role of economic evaluation in each case.

**Table 1:** Characteristics of case examples for evidence-based practice financing strategies

Characteristic	Case 1	Case 2	Case 3
**Financing strategies used**	Pay-for-Success	Alternative payment models: case management payment, pay-for-performance	Fiscal mapping process (i.e., tailoring of strategies)
**Service sector**	Behavioral health	Medical/hospital	Primary care
**Clinical problem**	Serious behavior problems (e.g., conduct disorder)	Cancer	Opioid use disorder
**Evidence-based practice(s)**	Multisystemic therapy (MST)	24/7 clinician access, patient navigation, comprehensive care plan, guideline-consistent treatment	Integrating Support Persons In Recovery (adapted Community Reinforcement and Family Training)
**Service modality**	Family- and community-based psychotherapy	Bundle of care coordination and treatment services	Group intervention for support persons; in-person and telehealth versions
**Other implementation strategies used**	Required readiness, training/consultation, and quality assurance activities	Audit-and-feedback, continuous quality improvement, updated health record systems	Training and ongoing supervision
**Evidence base**	Extensive clinical and economic effectiveness data	Extensive clinical data and more limited (but still promising) economic data	Original intervention has shown clinical efficacy; adaptation is being evaluated in a pragmatic trial
**Stakeholders involved in financing decisions**	Colorado Governor’s office; social impact investor companies; MST intermediary	Center for Medicare & Medicaid Services, based on diverse stakeholder input	Bottom-up decision-making: intervention developer conducts fiscal mapping with input from health system and funding agency partners
**Role of economic evaluation in financing decisions**	Projected cost-savings were the basis of selecting MST for implementation	A simulation budget impact analysis was conducted for the new payment models based on prior studies	Cost data are being collected and evaluated during the pragmatic trial

### Case 1: Pay-for-Success for multisystemic therapy

The first case is a Pay-for-Success financing initiative (also known as “Social Impact Bonds”) designed to address youth detention and incarceration through improved behavioral health services in Colorado. Detention and incarceration remain common, albeit largely ineffective, responses to serious youth behavioral problems in the USA [29, 30] and Colorado specifically [31]. Behavioral health EBPs can address risk for ongoing behavioral problems and reduce criminal recidivism, offering alternatives to youth detention and incarceration, yet only 1–3% of the eligible population receives these interventions [32].

In 2018, the Colorado Office of State Planning and Budgeting, in coordination with other state agencies and outside experts, chose to implement a behavioral health EBP called multisystemic therapy (MST [33]) as one of the state’s inaugural Pay-for-Success projects. MST is an intensive family- and community-based psychotherapy intervention for youth ages 12–17 at high risk for ongoing justice system involvement. Many features make MST an ideal candidate for Pay-for-Success financing [34]. MST addresses a key public health need (youth recidivism), and it is a well-defined intervention with numerous rigorous evaluations demonstrating immediate and long-term effectiveness [33, 35]. Important MST outcomes such as recidivism and out-of-home placements can be regularly tracked at the local or state level to document success. Finally, economic evaluations estimate that MST produces over $3 in economic benefits per $1 spent within 2 years post-treatment [36, 37].

Complex economic considerations limit availability of MST, despite strong evidence of its economic benefits from reduced recidivism and out-of-home placements [38]. For example, community-based behavioral health agencies that deliver MST pay up-front for implementation, often with support from juvenile justice partners, whereas Medicaid and adult criminal justice realize most of the eventual benefits from decreased arrests and out-of-home placements [36]. The per-patient costs of delivering MST are substantial, typically ranging from $8000–13,000 per 4–6 month treatment episode, as the intervention requires intensive services, small caseloads, and ongoing training, consultation, and quality assurance activities. Further, initial implementation costs related to readiness planning, training, and lost productivity are often a disincentive for behavioral health agencies to adopt MST. Novel financing solutions are needed to help states more widely implement this EBP while managing its cross-sector costs and benefits [34]. Pay-for-Success financing is one strategy for balancing the financial risks associated with EBP implementation, by enabling a government to partner with private investors such as individuals, foundations, or corporations—a prime example of coordinating financing strategies among funders (see key decision 4 in Fig. 1).

The Pay-for-Success financing strategy followed several key steps. First, the government prioritized a social problem and determined a solution to implement; the State of Colorado sought to decrease youth detention, incarceration, and recidivism by implementing MST in regions underserved for youth behavioral health. Second, private investors interested in achieving social impact provided the upfront capital for implementation. One bank (Northern Trust Corporation) and two foundations (Gary Community Investments, the Denver Foundation) were the investors for MST. The Center for Effective Interventions at the University of Denver was chosen as the intermediary organization for MST to provide implementation support to the community-based behavioral health agencies delivering the intervention. The investors, together with the Colorado State Office of Children Youth and Families and the Center for Effective Interventions, were appointed voting members of the Governance Committee overseeing the project. The Committee developed plans for an independent evaluator (Colorado Evaluation and Action Lab) to measure outcomes across 3 years of active MST implementation plus 1 year of follow-up evaluation. Finally, the Committee developed plans for the state government to repay the investors through “success payments” if the project meets key implementation outcomes (e.g., fidelity benchmarks correlated with long-term outcomes [33]) and youth outcomes (e.g., less time in secure detention or out-of-home placement, based on quasi-experimental propensity score matching), with larger payments for more favorable outcomes. The Committee may consider early termination if early outcomes indicate the project is unlikely to succeed. Importantly, Pay-for-Success shifted the financial risks of implementation from service and intermediary organizations to private investors, who could better afford to take such risks for the public good.

The substantial cost-benefit tradeoffs of MST were integral to supporting the financing arrangements for this project. Based on published evidence of >$3 for each dollar spent [36, 37] and the state’s predicted 28% reduction in youth recidivism [39], state-level savings for this initiative were conservatively predicted at over $8 million across 3 years. Implementation costs were the primary barrier to MST dissemination in Colorado, and Pay-for-Success financing offered a viable strategy to raise the upfront capital, share risk across public and private investors, and enable agencies to establish sustainable MST teams that may continue to produce a return on investment in the years ahead. The major drawback of Pay-for-Success is its complexity and scope, requiring almost a year of negotiation to establish all necessary financing, service provider, and evaluation agreements.

### Case 2: alternative payment models for oncology care

Over 1.6 million people are diagnosed with cancer annually in the USA, and almost 600,000 die from it, making it the second greatest cause of death [40]. The cost of cancer care has grown dramatically and was projected to reach $173 billion by 2020 [40]. In July 2016, the US Centers for Medicare and Medicaid Services launched the 5-year Oncology Care Model (OCM), its first major initiative to pilot the transition from fee-for-service payments to value-based purchasing in oncology for Medicare insurance beneficiaries [41]. The OCM is based on 6-month episodes of care for patients who receive chemotherapy. The model was developed partly to address the rising costs of oncology care [42] and the significant regional variation in quality and costs [43]. Medicare used economic analyses to design a novel payment strategy intended to promote delivery of higher-quality oncology care at the same or lower cost.

The oncology care EBPs required by the OCM were selected based on extensive clinical and economic evidence; for example, patient navigation services and expanded access to clinician experts have been shown to decrease the utilization of low value and avoidable acute care [44]. The goal of OCM was to design a financing strategy that was ideally suited to supporting those EBPs (Fig. 1, key decision 3). To promote the use of EBPs, the OCM requires practices to provide the “enhanced services” defined as follows: 24-h/7-days-per-week access to a qualified clinician, patient navigation, a comprehensive care plan, and treatment consistent with nationally recognized clinical guidelines [41, 44]. Additionally, oncology practices must participate in audit and feedback with the Centers for Medicare and Medicaid Services and use federally certified electronic health records. The provision of “between office visit” care (e.g., patient navigator, care planning) is not funded by fee-for-service reimbursements, and 24/7 access to providers is typically underfunded [44], so incorporating these EBPs into the OCM reimbursement structure helps to support their sustained use. In contrast, traditional fee-for-service payments incentivize fragmented, high-volume services and increased costs of care, through failing to reward providers for improving care coordination, quality, patient experience, and reducing costs [41].

To address the financing barriers to oncology EBP use, the OCM builds on fee-for-service payments with two additional payment mechanisms [41, 44]: (1) a per-beneficiary $160 Monthly Enhanced Oncology Services case management payment paid prospectively throughout an episode and (2) performance-based incentive payments (i.e., a form of pay-for-performance) paid retrospectively on a semiannual basis [44]. The selection of the OCM’s payment mechanisms were informed by two successful demonstration projects of alternative payment and care-delivery models in oncology, though such analyses remain rare and do not always demonstrate improved benefits for the cost. Previously, the Community Oncology Medical Home care-delivery model showed reductions in emergency service visits and inpatient hospitalizations across seven US oncology practices [45]. Secondly, an episode-based payment model demonstrated 34% cost savings by aligning payment incentives with EBPs and reimbursing for enhanced care as compared to a fee-for-service system [46]. These demonstrations suggested financing strategies could be used to increase adoption and sustained use of cost-effective EBPs in oncology practices. Based on an extensive process of stakeholder input, the OCM was developed as a hybrid model combining the patient coordination elements of a medical home, the aligned financial incentives of an episode-based model (case management and incentive payments), and a familiar fee-for-service structure for other services [42].

After stakeholder input but before the structure and amounts of the proposed case management and incentive payments were finalized, Centers for Medicare and Medicaid Services conducted a simulation budget impact analysis of OCM for Medicare and oncology practices [47]. To develop the $160 monthly case management payment ($960 per 6-month episode), the analysts used the US Bureau of Labor Statistics wage estimates to model the costs for the additional staff and spread the costs across the average number of episodes per practice [48]. The performance-based payments are linked to quality measures and total cost of care per episode, incentivizing physicians to reduce costs while sustaining higher-quality care [41, 44]. Practices have the option to choose between two payment models: (1) can receive incentives but are not financially penalized for failing to meet performance targets (one-sided risk to payors); and (2) can earn larger incentives for meeting more stringent performance metrics, but also face the risk of financial penalties (two-sided risk to providers and payors) [41]. The budget impact simulation found that Medicare could break-even on case management payments if practices reduced other service utilization costs by roughly 4%, which was deemed feasible [48]. In addition, the simulation revealed challenges of creating statistically reliable benchmarks for determining performance incentives, so the OCM may only work well for larger practices (>100 episodes per year).

As of February 2020, the OCM included 139 participating practices, over 6000 practitioners, approximately 25% of Medicare fee-for-service chemotherapy-related cancer care (>150,000 beneficiaries per year), and 10 non-Medicare payors [44]. As oncology practices adopt the OCM, it will now be important to evaluate its performance in terms of both EBP implementation and financial viability.

### Case 3: fiscal mapping process for a novel opioid use disorder intervention

This last case highlights how selection of financing strategies is also an important consideration during intervention development. The US opioid use disorder (OUD) epidemic has resulted in widespread impairment, death, and economic impacts [49]. Medication treatment for OUD (e.g., buprenorphine) effectively reduces OUD symptoms and overdose deaths, yet it remains underutilized, and patients often drop out of medication treatment [50, 51]. Greater engagement of patients’ support persons (i.e., partner, parent, other family member, or close friend) in the care process could increase initiation and retention with OUD medication [52, 53]. INSPIRE (Integrating Support Persons Into Recovery) is a novel approach to working with support persons of patients with OUD. INSPIRE is an adapted version of Community Reinforcement and Family Training, an evidence-based intervention that teaches support persons how to support a loved one’s engagement in substance use disorder treatment [54]. INSPIRE has a specific focus on supporting retention in medication for OUD, and includes ten 90-min group sessions (with rolling admission) covering topics of self-care, behavioral strategies, and relationship-promoting strategies. The INSPIRE developers are working with primary care systems and funding agency representatives to evaluate its clinical and economic impacts in a pragmatic clinical trial [55]. In response to the COVID-19 pandemic, the developers are also developing a telehealth-format version called eINSPIRE.

Despite its strong foundation, INSPIRE could prove challenging to implement even if it improves MOUD retention. Family treatments for substance use disorders have low uptake in service systems, due to financing barriers for interventions that focus on people other than the identified patient [56] and the limited economic evaluations of such treatments [57]. To address these cost- and financing-related barriers, the INSPIRE pragmatic trial deploys a recently developed fiscal mapping process to identify key intervention components and link them to sustainable financing strategies (see [24]). Fiscal mapping is a five-step process adapted from Intervention Mapping, a well-established method recommended for tailored selection of implementation strategies [58]. The steps of fiscal mapping help health service organizations to identify financial investments needed to maintain components of an EBP (e.g., staff time, supplies) and associated implementation strategies (e.g., ongoing training, interagency partnerships), then match those needs to appropriate financing strategies. Thus, the process can guide decisions around selection and coordination of financing strategies to support EBP sustainment (i.e., key decisions 3 and 4 in Fig. 1). Thus far, the INSPIRE team has:

1. Conducted an assessment of resources needed to sustain INSPIRE (e.g., staff time, training and supervision, telehealth equipment)
2. Specified funding objectives (e.g., cover provider time for delivering INSPIRE, cover INSPIRE training costs) and related determinants for each resource (e.g., whether a given expense is allowable for certain settings, funders)
3. Identified candidate financing strategies (e.g., fee-for-service reimbursement, grant/contract arrangements, build into internal budgets) for each funding objective

These activities have identified Medicaid billing options that could most sustainably finance INSPIRE groups and a need for private funding to purchase technology that support persons can use to participate. The remaining steps are to (4) work with health system partners (including financial and accounting staff) and funding agency partners to track INSPIRE implementation costs and help them select financing strategies (from step 3) that sustainably cover those costs; then, (5) monitor and evaluate financial viability over time. The result will be a comprehensive fiscal map outlining how INSPIRE can be financially sustained in primary care systems—should the pragmatic trial show it produced significant clinical benefits for its costs.

Fiscal mapping offers a unique approach to financing decisions for new interventions like INSPIRE. Typically, health service providers offer services for which funding agencies are willing to offer payment [23] (see also Fig. 1). Intervention developers create effective interventions, then seek to convince funders to pay for them [59]. The INSPIRE team has taken a more collaborative approach that involves all stakeholder groups—intervention developers, health systems, and funding agencies—working together from the beginning to plan for the complex realities of financing INSPIRE’s implementation. If sustained use and scale-up prove desirable for INSPIRE, the developers will also have knowledge of feasible financing strategies, which could accelerate its uptake by service providers.

## Discussion

A major challenge to implementing and sustaining EBPs is covering associated costs with the limited, fragmented funding available [5, 23, 24]. Our case examples show how financing strategies may overcome cost-related barriers for EBPs by organizing stakeholders around complex interventions (case #1), optimize the clinical and economic benefits of an intervention package (case #2), and link available funds to financing needs (case #3). Overall, financing strategies help translate findings from economic evaluation research (What should we fund?) into policy and practice (How should we fund it?), enabling the public health and economic impacts of EBPs. However, financing strategies remain poorly understood [24], and additional efforts are needed to align funding with EBP implementation and sustainment needs. Therefore, we suggest a research agenda addressing five key issues related to financing strategies.

First, as articulated elsewhere [9], maximizing the relevance of economic evaluations to real-world EBP use can help translate findings into practice. Economic evaluations of EBP implementation or implementation strategies will continue to be important (e.g., [9, 36]; see also key decisions 1 and 2 in Fig. 1), but scholars increasingly warn that implementation research often does not represent typical practice [60]. Rather than developing and testing highly complex, multi-component implementation strategies to maximize between-group effect sizes, we can pursue a more pragmatic approach that prioritizes lean implementation, efficient use of scarce resources, and usability by providers and provider organizations [61]. Stakeholder involvement and consideration of financing throughout the research process is critical to avoid the development of implementation strategies that are too complex, and therefore too expensive, to fund in practice. Moreover, the fiscal mapping process from case #3 could be used to link funding sources to each EBP or implementation strategy in ways that enhance implementation outcomes. Such research will need to incorporate theories and methods from beyond implementation science and health economics—including market access [27] and organizational financing theories such as resource dependence [28, 62] or open systems [63]—as well as financing and accounting professionals as research partners.

Second, changes in real-world financing systems can inform future economic evaluations. Health systems are being transformed by the needs of chronic disease management, health promotion, management of health risks at a population level, and paying for outcomes (vs. fee-for-service) [64]. As payors adopt new financing mechanisms for healthcare, economic evaluations will need to reexamine traditional assumptions about measuring costs and highlight the main cost and benefit drivers in implementation strategies and EBPs. For example, the OCM payment model (case #2) assumes that shifting financial incentives from the payor to the provider will result in the successful adoption of EBPs by practices and use with patients. Payors will be most interested in whether the OCM encourages high-quality care while containing their own costs, but providers could experience substantial unreimbursed costs of implementing and sustaining EBPs [65]. Practice-based research could help identify key drivers of those costs (e.g., provider training, operational changes to workflows)—including qualitative or mixed-method research when cost implications are initially unclear [8]. Moreover, working with health systems can help identify financing-related barriers to de-implementation (e.g., perverse incentives to provide low-value services that are profitable) that are important to consider when understanding the economic impacts of EBP implementation on systems [66].

Third, it will be challenging to evaluate which financing strategies are most effective and economically beneficial under a given set of conditions. It remains unclear what level of evidence (e.g., from economic evaluations) justifies a given level of financial risk and who should bear that risk. Many financing strategies involve coordinating funds across multiple levels of the service system ecology (e.g., fiscal mapping of provider, clinic, and health system funding needs) and/or across multiple service systems (e.g., juvenile justice, behavioral health, and private investors in case #1). Designing these strategies is technically complex and requires expertise from finance, accounting/billing, and economics professionals. For example, cost-sharing among the parties involved is a challenging aspect of financing strategy design; healthcare reforms increasingly shift costs onto providers, who risk lower reimbursements or even penalties for not achieving quality standards or desired outcomes [67]. It will also be important to explore how strategies that incentivize EBP use (e.g., Pay-for-Success, performance incentives) work in diverse health systems, for example, many commercial payors have oncology patient populations that are too small to perform risk adjustment, limiting alternative payment models’ feasibility [68]. Moreover, it is important to consider patients’ active roles in financing care: to what extent is it feasible, equitable, and ethical to use co-pays or other patient payments to fund EBPs? To the extent possible, financing strategies should be straightforward and target the level(s) or system(s) that produce the largest improvements in implementation outcomes; complex strategies may require their own implementation support, as considerable challenges to execution have been observed [69, 70].

Fourth, the impact of financing strategies should be investigated across pre-implementation, active implementation, and sustainment phases [71]. Almost all implementation research involving financing strategies [24] or economic evaluations [2, 3] has focused on active implementation, yet all phases are relevant to financing decisions. Pre-implementation involves considerable costs (e.g., engagement and readiness planning [72]) that are rarely reimbursed. Sustainment is also critical because the ultimate public health impact of EBPs is dependent on long-term use [73]. The initial OCM modeling did not separate implementation versus sustainment costs, and a professional organization has advocated that much higher monthly case management payments ($225–675) could be necessary to achieve financially sustainable offsets in service utilization [74]. Research on economic impact and financing strategies across implementation phases can help guide decisions about when and how to invest in implementation. Furthermore, longitudinal research will be useful for examining how the use of financing strategies unfolds dynamically across implementation phases. In Colorado, discussions are ongoing about whether the Pay-for-Success investors will re-commit their payouts to support sustainment of MST, or if their initial funding should be used as a “bridge” to other long-term financing strategies.

Finally, researchers need to navigate relationships among health service organizations and their funding sources (see Fig. 1) when studying EBP financing. Most organizations have limited control over the financing strategies available to them, though they may engage in strategic decisions or negotiations about which they use [75, 76]. For example, a community oncology clinic might need to participate in multiple alternative payment models so that it can cover all its cancer patients, and could advocate for changes in reimbursement amount and structure that make such arrangements more feasible. However, there remains considerable information asymmetry between payors and providers in the healthcare marketplace: Providers may resist sharing the true costs of care to avoid scrutiny from funders, and commercial funders consider payment models proprietary and tend to only release details when the payment model showed economic benefits [25]. These challenges compound widely recognized barriers to providers’ sharing patient and service utilization data (e.g., privacy issues, limited capacity). Overall, research on financing strategies requires the ability to develop strong, trust-based partnerships with stakeholders, and support stakeholders in partnering with each other. Our Pay-for-Success and fiscal mapping process case examples portray such partnerships and further suggest that transparency in price structures are critical to the development of financing strategies that can truly impact implementation outcomes.

It is important to note several limitations of our analysis. We presented case examples related to EBP treatment models, but it will also be important to explore the challenges of financing of EBPs that are preventive interventions, screening/assessment tools, or policy solutions—where returns on investment tend to be diffuse and unfold slowly over time [77]. Further, we are US-based researchers, and presented cases from the US. Global health research offers numerous opportunities to compare varied approaches to EBP financing [18, 78, 79]. Finally, an in-depth analysis of how the COVID-19 pandemic has affected EBP financing would be useful to investigate how financing strategies respond to “shocks to the system,” whereas we described how strategies function under more stable conditions.

## Conclusions

In conclusion, EBP financing strategies offer promising solutions for translating economic evaluation results into policy and practice, but raise numerous questions. Those questions illustrate the various dimensions of interventions, systems, and broader contexts that will be important to consider as researchers and stakeholders develop a robust field of scholarship around EBP financing strategies.

## Data Availability

The datasets supporting the conclusions of this article are available upon request through the corresponding author.

## Abbreviations

EBP: Evidence-based practice
INSPIRE: Integrating Support Persons Into Recovery
MOUD: Medication for opioid use disorder
MST: Multisystemic therapy
OCM: Oncology Care Model
OUD: Opioid use disorder
